# Variation in ambulance pre-alert process and practice: cross-sectional survey of ambulance clinicians

**DOI:** 10.1136/emermed-2023-213851

**Published:** 2024-12-05

**Authors:** Joanne E Coster, Fiona C Sampson, Rachel O'Hara, Jaqui Long, Fiona Bell, Steve Goodacre

**Affiliations:** 1 The University of Sheffield, Sheffield Centre for Health and Related Research (SCHARR), Sheffield, UK; 2 Yorkshire Ambulance Service NHS Trust, Wakefield, UK

**Keywords:** pre-hospital, emergency ambulance systems, emergency care systems, guideline, communication

## Abstract

**Background:**

Ambulance clinicians use pre-alert calls to inform emergency departments (EDs) about the arrival of critically ill patients. However, there is variation in guidance between local ambulance service policies in terms of what should be pre-alerted and how pre-alerts should happen. We conducted a national online survey to understand the use of ambulance pre-alerts and to inform recommendations for practice and guidance.

**Methods:**

Ambulance clinicians in England involved in pre-alert decision-making were recruited via ambulance trusts and social media to complete an anonymous online survey conducted during May–July 2023. Quantitative data was analysed descriptively using SPSS (version 28) and free-text responses are reported to illustrate the quantitative findings.

**Results:**

We included 1298 valid responses from 10 English ambulance services. There was variation in practice at all stages of the pre-alert process, including the reported frequency of pre-alert (7.1% several times a shift, 14.9% once/two times per month). Most respondents reported that pre-alerts were delivered directly to the ED, but 32.8% reported pre-alerting via an ambulance control room. A third of respondents always used mnemonics to guide a pre-alert (eg, ATMIST (Age, Time of Incident, Mechanism of injury, Injuries, Signs, Treatments)), but 10.2% reported not using any fixed format.

The type of guidance used to identify patients for pre-alert varied between clinicians and ambulance services, with local ambulance service guidance being most commonly used, and 20% stating they never use national guidelines. Respondents reported variable understanding of appropriate conditions for pre-alert, with paramedic students particularly wanted further guidance on trauma in older patients and medical pre-alerts. 29% of respondents reported receiving specific pre-alert training, while 50% reported never receiving feedback.

**Conclusion:**

We identified variation in pre-alert processes and practices that may result in uncertainty and challenges for ambulance clinicians providing time-critical care. Guidance and training on the use of pre-alerts may promote more consistent processes and practices.

**WHAT IS ALREADY KNOWN ON THIS TOPIC**

- Pre-alerts can enable emergency departments to prepare for the arrival of a critically ill patient; however, there is variation in local ambulance trust pre-alert guidance and in the patients and conditions that are pre-alerted. The reasons for this are unclear.

**WHAT THIS STUDY ADDS**

- This survey of ambulance clinicians in England found variation in reported practice in how pre-alerts are delivered across ambulance services and between individual clinicians.
- The study identifies a lack of formal training and feedback around pre-alerts and that a majority of ambulance clinicians would find additional training and feedback useful.

**HOW THIS STUDY MIGHT AFFECT RESEARCH, PRACTICE OR POLICY**

- Guidance and additional training in the use of pre-alerts could promote more consistent processes and practices.
- Further research is needed to evaluate methods for providing feedback and to understand the impact of additional pre-alert training, guidance and feedback in improving pre-alert practice and increasing consistency.

## Introduction

 Pre-alerting by ambulance clinicians to the destination emergency department (ED) is a key part of the emergency care process for critically unwell or injured patients who may require time-critical treatments and swift senior clinical review. A pre-alert involves contacting the ED by telephone ahead of the patient’s arrival to provide information about the patient, allowing EDs to prepare staff and allocate appropriate space such as a resuscitation area. In England, more than 1 in 10 ambulance conveyances are pre-alerts, but routine data show variation in the patients and conditions that receive pre-alerts.^1^

Previous research identified beneficial evidence of pre-alerts for stroke, trauma and sepsis. Benefits for stroke include shorter door-to-needle times, shorter time to receiving a CT scan and an increase chance of receiving thrombolysis within the therapeutic window^2^; benefits for trauma include improvements in resuscitation readiness and performance^3^; and for sepsis, benefits include faster administration of fluids, tests and antibiotics.^4^ However, Sheppard *et al* identified variability in pre-alert practice for stroke, including pre-alerting patients who did not meet protocols and guidance for pre-alerting, different interpretations of pre-alert protocols between ED and ambulance staff, disagreements over pre-alert practice and negative feedback from the ED about pre-alert decisions.^4^ Overuse of pre-alerts or perceived inappropriate pre-alerts may result in pre-alerts receiving a reduced response (pre-alert fatigue). A review of UK ambulance service pre-alert guidance identified local service variation in pre-alert thresholds compared with national guidance, potentially leading to local service variation.^5^ The review also highlighted inconsistent terminology and methods used for pre-alerting, which may cause confusion and miscommunication of pre-alerts.^5^ There is some evidence that the use of structured communication methods, such as Situation, Background, Assessment, Recommendation (SBAR), a format used to communicate pre-alerts, has been found to improve patient safety.^6^

In the USA, an evidence review identified that pre-alerting (or pre-notifying) the receiving facility has the most promising level of evidence for improving stroke care. Some US states, (eg, Wyoming) require pre-alerts for patients meeting stroke criteria by law and recommends other states follow this example.^7^ In England, the Ambulance Association of Chief Executives (AACE) and the Royal College of Emergency Medicine (RCEM) issued joint pre-alert guidance in 2020.^8^ However, there is still significant variation in pre-alert guidance across different ambulance services, with ambulance service guidance varying from the national AACE/RCEM guidance in most UK trusts.^9^

This research forms part of a mixed-method study exploring pre-alert decision-making, communication and the impact of pre-alerts on receiving EDs and patients. In this paper, we present survey results from ambulance clinicians to describe their decision-making and communication experiences with pre-alerts. We also examine differences in pre-alert processes across clinician types within services.

## Methods

We conducted a cross-sectional online survey nested within a larger mixed methods study.^10^

### Sampling and recruitment

We recruited ambulance clinicians from ambulance trusts in England via local ambulance trusts. We define ambulance clinicians as follows: paramedic, advanced/specialist paramedic, student paramedic and emergency medical technicians and any other ambulance personnel responsible for the delivery of a pre-alert. Ambulance trusts used their usual staff research recruitment methods including emails, newsletters, staff social media groups, posters and advertising on Twitter (now called X). These methods differed by site.

### Mode of administration

The survey, conducted online using Qualtrics, was accessible via a weblink or QR code from 1 May to 14 July 2023, for at least 6 weeks at each site. Participants were required to confirm their understanding of the study, that they are an ambulance clinician involved in pre-alert decision-making and provide their consent prior to accessing the full survey. The survey access link was sent to each site once research governance approval had been obtained. The survey was accessible from various electronic devices, including mobile phones, laptops and tablets. At the end of the survey, participants were given the opportunity to anonymously enter a prize draw to win a £50 voucher.

### Survey development

The questionnaire was developed based on the literature and preliminary analysis of interviews with ambulance clinicians. Survey questions explored the pre-alert process from decision to pre-alert to ED response^11^ (see table 1). Information on respondent characteristics was collected to examine results by service and role.

**Table 1:** Survey topics

Section 1: Making a pre-alert decision	Reasons for making a pre-alert. Frequency of making a pre-alert. Actions when unsure whether to make a pre-alert. Guidance used to aid pre-alert decisions. Factors affecting decisions to pre-alert. Areas where more pre-alert guidance would be useful.
Section 2: Undertaking the pre-alert call	Who contacts the receiving ED. What device is used to contact the receiving ED. Is the pre-alert recorded in the patient notes and if so how is it recorded. Learning to make a pre-alert. Feedback on pre-alert decisions.
Section 3: Communicating with the ED	ED staff responses to pre-alert calls (taking pre-alerts seriously, making appropriate plans in the department, listening without interrupting). Pre-alert format used.
Other	Anything else to add about pre-alerts

EDemergency department

Survey questions used rating scales, multiple and single choice tick boxes and text boxes to provide additional information.

An initial survey draft was developed by the research team and piloted with ambulance clinicians from different ambulance services. We used the survey pilot to develop a questionnaire that was relevant to and inclusive of all ambulance services. We received 13 responses to the survey pilot. The survey was also reviewed by each ambulance service trust as part of the local research approval process, which generated additional survey feedback, resulting in some changes to the survey, including a reduction in the number of questions. The final survey, containing 16 questions, was approved by local ambulance service trusts and NHS ethics. A copy of the survey is provided in online supplemental appendix 1.

The survey did not collect identifiable information. Participants could enter a survey prize draw by accessing a separate form and entering their email address. This information could not be linked to survey responses.

### Analysis

Survey data was collated in the Qualtrics platform and analysed in SPPS V.28.^12^ Partially completed surveys, defined as <70% completion, were excluded from the analysis. Variables were cleaned and modified to facilitate analysis. Categorical data were assigned a numerical value and labelled and responses reported at the number and proportion in each category. Likert scale ratings were treated as continuous data and reported using the mean, SD and proportion of responses at the scale endpoints (always or never). Missing values were coded as missing.

A descriptive analysis of the responses to the 16 survey questions and of respondent characteristics was undertaken in SPSS to identify the frequency and range of responses. Variation was explored through subgroup analyses using ambulance service and staff role variables.

Free-text responses were extracted into Microsoft Excel and used to further understand experiences and views of the pre-alert process. Comments that most succinctly represent the themes and issues in the data and a range of ambulance services are included as supporting information in the analysis.

### Patient and public involvement (PPI)

A diverse PPI group of patients and carers with lived experience of the pre-alert process reviewed and commented on survey questions and processes. Project management meetings to refine and develop the survey were attended by PPI representatives. Results were presented at a PPI workshop and PPI views of the findings were discussed.

## Results

1641 responses to the survey were received and 343 were excluded. Reasons for exclusion were: missing data, n=298; responses from private providers n=18; responses from devolved countries n=27. This left 1298 complete responses from English ambulance services included in the analysis.

Table 2 reports respondent characteristics, and online supplemental table 1 describes respondent characteristics by service. Response rates varied by service. Two services (4 and 6) did not have capacity to promote the survey internally resulting in a lower response. Over 50% of the sample were paramedics, with a further 14% being specialist or senior paramedics. Almost half (45%) had over 6 years in their role. Most respondents were white males 56.5% (n=734), or white females 36.5% (n=473), which reflects the NHS workforce survey ethnicity profile.^13^

**Table 2:** Respondent and workforce characteristics

Role	ParamedicN (%)	Specialist or senior paramedicN (%)	Emergency medical technician, or equivalentN (%)	OtherN (%)	TotalN (%)
All respondents	688 (51.5)	179 (13.8)	307 (23.7)	39 (3.0)	1298 (100)
**Length of time in role**	**<2yearsN (%)**	**2–5yearsN (%)**	**6–10yearsN (%)**	**>10yearsN (%)**	**TotalN (%)**
All respondents	291 (22.4)	430 (33.1)	318 (24.5)	256 (19.7)	1298 (100)
**Gender**	**FemaleN (%)**	**MaleN (%)**	**Non-binaryN (%)**	**Other/prefer not to sayN (%)**	**TotalN (%)**
All respondents	492 (37.9)	768 (59.2)	10 (0.8)	20 (1.4)	1298 (100)
**Ethnicity**	**WhiteN (%)**	**Other ethnic groups combinedN (%)**	**Prefer not to sayN (%)**	**–**	**TotalN (%)**
All respondents	1224 (94.3)	41 (3.1)	27 (2.0)	–	1298 (100)

### Making a pre-alert decision

Table 2 reports reasons for making a pre-alert call and factors impacting these decisions. Online supplemental tables 2 and 3 report pre-alert practice by ambulance service and clinician’s reasons for making pre-alert calls. Clinicians mostly reported making pre-alert calls to inform the ED of a deteriorating patient or to make space in the resuscitation. Phoning for advice about where to take the patient was the least common, though in some services, over a third of staff always used pre-alerts for advice. Text comments identified other reasons for pre-alerts: informing receiving hospital of additional needs, for example, translation services, mental health, infectious patient; to comply with protocols; requesting specialists; warning of violent or difficult patients; ambulance clinicians also pre-alerted when patients were too sick to queue but did not require resuscitation.

The patient doesn't need resus but cannot be at the back of an 11 patient queue in the corridor. They need rapid assessment and triage, though not necessarily rapid treatment in resus (Paramedic, 2 – 5 years’ experience Service 1)

The survey asked which sources of guidance do you use to help you decide whether to make a pre-alert (Q4), and identified a range of guidance sources (table 3). Local ambulance trust guidance was identified as most used and this was consistent across different types of ambulance clinicians. There was variation in the use of the national AACE/RCEM guidance^8^; overall analysis showed a fifth of ambulance clinicians never using this guidance; however, this ranged by ambulance service from 3.9% to 29.2%. Text comments identified differences in the pre-alert guidance and thresholds used by ambulance clinicians and those used by the ED, which sometimes led to EDs seemingly rejecting or responding dismissively to a pre-alert which met local or national ambulance pre-alert guidance.

**Table 3:** Types of patients where further guidance would be welcomed, by job role type

Further guidance	N (%) answering yes additional guidance would be useful				
	Paramedic	Specialist	Student	EMT	All staff
Trauma generally	346(51.8)	56(31.3)	66(64.1)	143(46.6)	639(49.2)
Silver trauma	456(68.3)	91(50.8)	76(73.8)	187(60.9)	842(64.9)
Medical pre-alerts generally	388(58.1)	80(44.7)	62(60.2)	188(61.2)	744(57.3)
Sepsis	273(40.9)	66(36.9)	51(49.5)	130(42.3)	541(41.7)
Respiratory	271(40.6)	48(26.8)	61(59.2)	122(39.7)	524(40.4)

EMTemergency medical technician

It’s infuriating when following specific guidance which dictates pre-alert but finding ED essentially not taking it seriously on your arrival (Paramedic > 10 years experience Service 5)

One of the biggest challenges is ambulance services and hospitals having differing views/policies on what would warrant a pre alert. There needs to be a clear, consistent criteria that both ambulance staff and hospitals follow. Sometimes, I am met by a poor attitude from staff in ED due to them thinking an alert is unnecessary even though it is within my guidance to make the alert. (Paramedic < 2 years experience Service 2)

I have found one of my local hospitals have staff who have different expectations about which patients we should pre-alert. We have pre-alerted a patient in Fast [Atrial Fibrillation] with a rate of 160 and been told by a consultant that he wasn't bothered if it wasn't over twice their age and then on another occasion I did not pre-alert a patient who was in AF with a rate of 110 and was treated as though I should have pre-alerted her. There should be more consistency in the staff in the hospitals. (Paramedic <2 years Service 9)

JRCALC [Joint Royal Colleges Ambulance Liaison Committee] guidelines are good and I try to follow these where possible. However I have had to learn that certain hospitals want pre alerts for other things that I have just had to figure out as I go along. (Paramedic < 2 years experience Service 5)

Table 4 also shows variation in pre-alert practice for clinical pathways where there is clear national guidance around making a pre-alert. Three quarters of clinicians reported always pre-alerting cardiac/respiratory arrest compared with under a quarter stating they alert for patients with tachycardia of ≥131 and respiratory rate of ≥25.^8^ Variation was identified in relation to pre-alert decision-making where there was no condition specific clinical pathway (table 4). Hospital destination had the most impact on pre-alert decision-making for all staff groups. Approaching end of shift was considered to have the least impact on pre-alert decision-making.

**Table 4:** Reasons for making a pre-alert call and factors impacting on pre-alert decisions

	Mean(1=never; 5=always)	SD	AlwaysN (%)	NeverN (%)
Reasons for making a pre-alert				
To inform ED staff of a potentially deteriorating patient	4.06	0.933	491 (38.2)	10 (0.8)
To give the ED time to make space in resuscitation	3.79	1.091	401 (31.7)	31 (2.5)
To ensure the patient is seen quicker on arrival	3.73	1.177	380 (31.9)	73 (6.1)
For advice about where to take the patient	2.50	1.218	55 (5.4)	268 (26.5)
Sources of guidance used to help make pre-alert decisions				
JRCALC	3.49	1.13	255 (19.6)	59 (4.5)
Local ambulance trust	3.75	1.06	338 (26.0)	36 (2.8)
Local hospital	3.10	1.25	140 (10.8)	149 (11.5)
ACCE/RCEM	2.87	1.27	91 (7.0)	202 (20.6)
Factors impacting on pre-alert decision-making				
Hospital transporting to	3.16	1.147	128 (9.9)	119 (9.2)
Distance from hospital	2.94	1.21	111 (8.6)	162 (12.5)
Anticipated Queue at the ED	3.00	1.19	100 (7.7)	151 (11.6)
Approaching end of shift	2.25	1.33	54 (4.2)	369 (28.4)
Physiological criteria or specific conditions that trigger you to make a pre-alert call				
Tachycardia≥131	3.54	0.99	234 (18.0)	19 (1.5)
Cardiac/respiratory arrest	4.54	0.90	966 (74.4)	2 (0.2)
Unconscious with a GCS motor score of less than 4	4.43	0.89	829 (63.9)	4 (0.3)
Respiratory rate=25	3.68	0.97	299 (23.0)	12 (0.9)

AACEAmbulance Association of Chief Executives EDemergency departmentGCSGlasgow Coma Score JRCALCJoint Royal Colleges Ambulance Liaison CommitteeRCEMRoyal College of Emergency Medicine

Text comments identified that pre-alert decisions were multifactorial and were also dependent on the requirements and varying guidance of local EDs.

Every ED seems to be different and there is a huge variation even between staff within the same ED to pre-alerted patients, which makes it seem like whatever you do/don't pre-alert, you are invariably in the wrong (according to them). I also think it is difficult with conditions such as sepsis, where if you follow the trust policy and pre-alert, you will get eye-rolled and no bed/quicker treatment/response for the patient, so it almost feels embarrassing doing the pre-alert but then it feels like there’s the risk of getting into ‘trouble’ from the ambulance trust if you don't stick to the policy they have written. (Paramedic > 10 years experience Service 10)

The challenge is not just the pre-alert process but also navigating which types of patients which hospitals want pre-alerted or not. For instance, in my area, one hospital has a fractured NOF pathway and want a pre-alert, but the other hospital doesn't, so don't want a pre-alert for fractured NOF patients. The local Tus [trauma units] will often tell you that you should take a trauma patient to an MTC [Major Trauma Centre] during the pre-alert call, despite the patient not meeting the local decision-tree criteria for MTC (Specialist paramedic 2- 5 years experience Service 7)

Survey question 7 asked ambulance clinicians whether they would find additional guidance useful and in what areas. Respondents indicated that in most areas, more guidance would be well received, particularly in older person trauma (65% would like more guidance); in medical pre-alerts 57% would like more guidance. table 3 reports these results by staff type.

The survey asked ambulance clinicians what they would do if they were unsure about whether to make a pre-alert call. Over half stated they would make the pre-alert call anyway, with another quarter stating they would call the pre-alert phone to discuss with the ED (table 4). Text responses identified a practice of making ‘courtesy calls’ or ‘heads up’ calls.

Either make a "courtesy call" to the pre alert phone to give them a heads up you’re coming, this [patient] could deteriorate but also could be absolutely fine, so when you turn up it’s not a massive surprise if they're on the edge of deteriorating (Paramedic 6 – 10 years experience Service 8)

### Pre-alert calls and processes

Online supplemental table 2 reports pre-alert practice by ambulance service and shows that over 80% of respondents reported making a pre-alert either frequently, often or once or twice a week (1061/1298) (online supplemental table 2). However, free-text responses showed that this was difficult to quantify due to the variability of patients seen.

Very much pot luck. You can have a run of shifts where every other job is a pre alert and others where you do none. (EMT, > 10 years experience Service 2)

Variation in how pre-alert calls came through to the ED was mostly service based. In over half of services (54.8%; 711/1298) common practice was for the ambulance clinician on scene to make the call to the ED, whereas in some other services standard practice was for the ambulance clinician on scene to phone through to the ambulance control desk, who would then call the ED pre-alert phone and pass on the information. Practice was sometimes different for medical and trauma calls.

The hardware for pre-alert calls also varied by service (online supplemental table 2). Ambulance radios were used infrequently in most services, with most calls made using personal mobile phones. Most respondents reported always recording the pre-alert in the patient notes and using a tick box plus free text; however, some variation by service was observed (online supplemental table 2).

### Learning how to make a pre-alert

Online supplemental table 2 also reports responses to questions 12 and 13, which asked ambulance clinicians how they learn to make a pre-alert and if they had ever received feedback on how they make pre-alert calls or decisions. Most respondents reported they had not received any specific training on how to make a pre-alert call (65.8%; 854/1298). Other, more informal training methods, were used, such as 59.2% (769/1298) reported learning from a mentor or senior colleague; 58.6% reported learning as they went along/on the job (761/1298); and 20.6% reported learning from written guidelines (267/1298). Most staff members (53.5% 695/1298) reported that they had never received feedback from either EDs or their ambulance service and this was consistent across most different services. Text comments highlighted the perceived usefulness of feedback, but cautioned that feedback was very negative for a perceived wrong pre-alert decision.

I was questioned by a clinician receiving the pre-alert on why I was pre-alerting a patient into hospital, despite a genuine clinical concern from ourselves for the patient. The person on the phone stated she thought it was an inappropriate pre-alert. (Student paramedic, 2 – 5 years experience Service 2)

As a graduate paramedic I received helpful feedback on every pre-alert I made as a student but I have received no feedback as a qualified paramedic. (Paramedic, 2 – 5 years experience Service 8)

The majority of the time I worry about pre-alerting too much and then worry about making pre-alert calls for pts I am unsure about. A feedback system would be greatly appreciated, as I have never received formal feedback from a hospital. I tend to base my pre-alert decisions on my own clinical judgement (need, observations, intervention, overall clinical picture, ongoing care, formal pathways etc.) in the hopes that this is appropriate. (Paramedic < 2 years experience Service 7)

I feel that the JRCALC guidance suggests a pre-alert for too many conditions, some staff follow this guidance. I know that at our local hospital, ED staff ask which trust you are from before deciding whether to action the pre-alert or not. (Paramedic 2 – 5 years experience Service 3)

### Communication with the ED

9% of ambulance clinicians felt that ED clinicians always listen and take the call seriously, always listen without interrupting and always make appropriate arrangements in the ED. There was little variation by role reported; however, student paramedics reported experiencing more interruptions on calls, while senior paramedics had the highest ratings for being listened to and taking the call seriously (see online supplemental table 4).

Often interrupted or questioned about my decision to pre-alert which takes time away from patient care. (Paramedic, 2 – 5 years experience Service 7)

ED staff often interrupt and do not fully listen and can sound dismissive. (Paramedic, > 10 years experience Service 10)

ED staff often lack insight into the fact we have very little bandwidth for the pre-alert. Often a paramedic is managing an acutely unwell patient and ED staff forget this. ED staff often interrupt and ask questions that can be better answered at handover or simple are not relevant at that time. (Paramedic, 6 – 10 years experience Service 2)

The seniority of the staff member who picks up the phone seems directly linked to how much they interrupt the pre-alert, i.e. a doctor will often just listen, a nurse will interrupt to fit the information in the order they are running through the list their side, which may differ to how the handover is being given. It is easier to pre-alert to a member of hospital staff that you already know because they trust your clinical judgement, as opposed to someone who does not know you. (Paramedic 6 – 10 years experience Service 6)

Local hospital will always try to interrupt, they are not trained in how to take a pre-alert. (Paramedic >10 years experience Service 9)

The format of the verbal communication with the ED varied overall and also by staff type. A third of ambulance clinicians reported always using a fixed format (35.7%; 464/1298); however, 1 in 10 reported always providing observations but not following a fixed format (10.2%; 133/1298). Specialist/senior paramedics had less agreement for using a fixed format and more agreement for using the format that the receiving ED uses and providing observations but not following a fixed format.

We serve several hospital[s] in my area - each hospital appears to have different pre-alert rules - this would determine which hospital receives a pre alert or not – (Paramedic, > 10 years experience Service 1)

We have a particular hospital that is notorious for not taking pre-alerts seriously. Last week I pre-alerted a patient with a NEWS 2 of 13 - red flag sepsis & reduced GCS. We had a travel time of 20 minutes yet the p/t was not placed into resus because ‘there are no nurses to watch him’ No doctor had been informed & the p/t placed onto a normal handover bed in ED where he deteriorated & was moved into resus 20 minutes after we arrived. (Paramedic, >10 years experience Service 3)

The destination is a key decision maker, some hospitals are better than others when taking pre alerts, some turn into a lengthy unnecessary conversation. As well the level of incivility experienced over the phone and on handover influence a decision to pre alert or not. (Specialist paramedic, < 2 years experience Service 1)

## Discussion

### Summary of key findings

This survey of ambulance clinicians throughout England demonstrated substantial variation in pre-alert practice and the ED response experienced. Some of this variation is service-based, most notably, who makes the call to the ED (crew on scene or clinical hub) and ambulance clinicians use of pre-alerts for different purposes, as highlighted in table 3. We also found service-based variation in guidance/use of checklists. In addition, there was variation in local ED policy around what should be pre-alerted and this sometimes conflicted with local ambulance guidance. This challenge may be exacerbated by the range of different types of ambulance pre-alert guidance that were reported as used and reported low usage of the national AACE/RCEM guidance. While national guidance on pre-alerts for cardiac/respiratory arrest was always followed by a majority of ambulance clinicians, it was not always followed in a quarter of cases. Most ambulance clinicians reported not receiving any specific training on how to make a pre-alert, call and over half had never received feedback about pre-alert decisions. Fewer than 1 in 10 ambulance clinicians perceived their pre-alert calls were always listened to without interruption, taken seriously, or that appropriate arrangements were made in the ED decisions.

### Comparison with other literature

The literature exploring how pre-alerts are undertaken and used is limited. In our mixed-method study, our analysis of routine data identified differences in pre-alert rates between ambulance services.^1^ Our survey identified differences in pre-alert practice, including reasons for pre-alert and Boyd’s review of local ambulance service pre-alert guidance identified significant differences in the guidance, which may in part be a contributing factor to variation.^5^

Findings related to lack of feedback about pre-alert decisions are consistent globally. In the USA, 45.5% of Emergency Medical Service (EMS) clinicians reported no feedback within 30 days,^14^ and in Canada, feedback is not part of routine practice.^15^ A UK qualitative study found EMS professionals strongly desire feedback noting its benefits for professional development and improving patient care.^16^ A systematic review in 2023 identified that feedback improves care processes including clinical decision-making, protocol adherence and documentation.^17^

### Limitations

While online survey methods have limitations in relation to response rate and potential bias,^18^ using this method allowed us to gain a national view of pre-alert practice. We obtained responses from 1298 respondents. The high number of responses and engagement shows that this is a salient issue for ambulance clinicians. Response numbers varied between sites, partly due to differences in site recruitment strategies. Despite this, eight trusts each returned over 95 responses. Respondents are representative of the skill mix and diversity of ambulance clinicians.^13^

We addressed non-response bias through engaging with ambulance clinicians to develop the survey questions and formats, optimising the survey for mobile devices, offering completion incentives and piloting the survey to ensure relevance to each ambulance service.

There is nevertheless high potential for response bias, with respondents having stronger views or more negative experiences being more likely to respond. Therefore, the views expressed in the survey may not fully reflect the experience of all ambulance staff. The potential for response bias does not detract from the ability of the survey to highlight important issues with the pre-alert process.

Internal validity of survey data can be affected by multiple survey submissions.^19^ We recruited via ambulance service research departments, checked IP addresses for duplicates and assessed response similarity and saved survey progress to prevent multiple submissions.

While the findings reflect participant reports rather than what happened, they align with routine data showing variation in practice that is unexplained by clinical need.^20^ Some variation may stem from service level variation. For example, mandated information recording policies may affect pre-alert documentation. In at least two services, the ambulance control room, rather than the on-scene crew, relayed pre-alert information to EDs.

### Implications of the results for policy and practice

The wide variation in pre-alert policies across ambulance and ED services suggests there is a need for more consistently aligned policies. Co-producing and embedding national policy at local levels and developing enhanced guidance for pre-alerting could improve consistency.

The survey indicated that ambulance clinicians would welcome feedback on pre-alert decision-making. However, feedback mechanisms that do not place additional workload on already busy staff are required. Comments revealed concerns about inconsistent or unprofessional responses to pre-alerts, which we further explore in our qualitative work for this study.^21^

The survey highlights multiple uses of pre-alerts, ranging from patients who require resuscitation, calls for advice and informing the ED of patients who are potentially too sick to queue. Further investigation into these uses is needed given the pressures on the ED system.

## Conclusion

We identified wide variation in pre-alert practice, and this was partly due to variation in pre-alert policies at ED and ambulance service level. Variation can result in challenges for clinicians involved in pre-alerts at a time when they are caring for time-critical patients. Introduction of training and feedback may lead to opportunities for learning and improving pre-alert practice at individual clinician and service levels (table 5).

**Table 5:** Communication with the ED

	Mean(1=never; 5=always)	SD	AlwaysN (%)	NeverN (%)	Paramedics	Specialist/senior paramedics	Student paramedics	EMTs
When making a pre-alert call to the ED, do you feel that ED staff								
Listen to you and take the call seriously	3.31	0.90	114 (8.8)	15 (1.2)	3.24	3.53	3.24	3.36
Listen without interrupting	3.09	1.04	115 (8.9)	59 (4.5)	3.04	3.11	2.83	3.26
Make appropriate arrangements in the ED	3.21	0.94	111 (8.6)	25 (1.9)	3.16	3.38	3.20	3.20
When you phone the ED, what format do you follow?								
Use a predefined format, for example, ATMIST, ASHICE, SBAR	4.00	0.98	464 (35.7)	16 (1.2)	4.12	3.74	4.04	3.85
Use a different predefined format, please state	2.56	1.31	26 (2.0)	129 (9.9)	2.20	3.16	2.61	2.73
Use the format that the receiving ED uses	2.92	1.31	90 (6.9)	170 (13.1)	2.70	3.24	3.14	3.07
Provide observations but do not follow a fixed format	3.16	1.24	133 (10.2)	111 (8.6)	3.08	3.36	3.27	2.53

ASHICEAge, Sex, History, Injuries/illnesses, Condition, ETA (estimated time of arrival at receiving hospital)ATMISTAge, Time, Mechanism, Injuries, Signs, TreatmentsEDemergency departmentEMTsemergency medical techniciansSBARSituation, Background, Assessment, Recommendation

## supplementary material

10.1136/emermed-2023-213851 online supplemental file 1

10.1136/emermed-2023-213851 online supplemental file 2

## Data Availability

No data are available. All data relevant to the study are included in the article or uploaded as online supplemental information.
