# Supplementary material for: Variation in ambulance pre-alert process and practice: cross-sectional survey of ambulance clinicians
Source: Emerg Med J. 2024 Dec 5;42(1):e213851. doi: 10.1136/emermed-2023-213851 (PMC11874312; doi:10.1136/emermed-2023-213851)
Supplement: online supplemental file 1 [file emermed-42-1-s001.pdf]

Appendix 1: Survey

Survey of Ambulance Clinicians

Start of Block: Default Question Block

National survey of ambulance clinicians
Pre-alerts are used by ambulance clinicians to inform Emergency Departments that a critically ill patient is on the way.
A pre-alert call is defined in this survey as "A pre-alert call from an ambulance clinician to a receiving hospital should be used to provide information about the patient that will enable the receiving Emergency Department or other clinical area to decide the most appropriate response." We are inviting you to take part in a survey that will help to understand the use of pre-hospital pre-alerts, and to inform recommendations for future practice. The survey should take around 10 minutes to complete and you can review your answers at any time before you press submit. You will not be asked to give any personal information and your responses will be anonymous. At the end of the survey, you will have the opportunity to enter a prize draw for a £50 Love2Shop voucher. There is a voucher for each ambulance service. You can enter by providing your email address, which will be logged separately from the information in the survey.
If you have any queries about the survey, please contact prealerts@sheffield.ac.uk or Dr Fiona Sampson on F.C.Sampson@sheffield.ac.uk Please read the information sheet, which can be accessed by clicking the link below:

Pre alerts participant information sheet

Pre alerts participant information sheet Please only complete this survey if you are an ambulance clinician involved in pre-alert decision-making. Please tick the boxes to confirm you have read the participant information sheet and that you are happy to participate in order to continue to the survey.

Have you read and understood the information sheet?

○ I have read and understood the information sheet (1)

Are you happy to participate in the survey?

○ I am happy to participate in the survey (1)

End of Block: Default Question Block

Start of Block: Block 1

Section 1: making a pre-alert decision

1. What are your reasons for making a pre-alert call to the ED?

	1 = Never	5 = Always

	1	2	3	4	5

To inform ED staff of a potentially deteriorating patient ()	
To give ED time to find space in resus for the patient ()	
For advice about where to take the patient ()	
To ensure the patient is seen quicker on arrival ()	
Other (please state) ()	

2. How often do you or your colleagues on scene make a pre-alert call?

○ Frequently e.g. several times a shift (1)

○ Often e.g. once per shift (2)

○ Sometimes e.g. once or twice a week (3)

○ Infrequently e.g. once or twice per month (4)

○ Never (5)

○ Other (please state) (6) ____

---

3. If you and your colleagues on scene are unsure about whether to make a pre-alert call, what would you be most likely to do? (tick one box only)

○ Make pre-alert call anyway (5)

○ Call ambulance control centre for advice (please give details about who you call below) (4)
____

○ Call pre-alert phone to discuss with ED (6)

○ Call ED on another line (7)

○ Other (please state) (8) ____

---

4. Which of the following sources of guidance do you use to help you decide whether to make a pre-alert decision?

	1 = Never	5 = Always

	1	2	3	4	5

Association of Ambulance Chief Executives (AACE)/Royal College of Emergency Medicine (RCEM) guidance ()	
JRCALC ()	
Local Ambulance Trust Guidance ()	
Local Hospital Trust Guidance ()	
Other (please state) ()	

5. For patients without a clearly defined clinical pathway (e.g. not MI, stroke or major trauma), which of the following would affect your decision to make a pre-alert to the ED:

	1 = Never	5 = Always

	1	2	3	4	5

Hospital transporting to ()	
Distance from hospital ()	
Anticipated queue at ED ()	
Approaching end of shift ()	
Other (please state) ()	

6. As a general rule, which of the following physiological criteria or specific conditions would trigger you to make a pre-alert call?

	1 = Never	5 = Always

	1	2	3	4	5

Tachycardia >=131 ()	
Cardiac/Respiratory arrest ()	
Unconscious with a GCS motor score of less than 4 ()	
Respiratory rate =>25 ()	
Other (please state) ()	
Other (please state) ()	

7. Please tick any areas where you would find additional pre-alerts guidance useful

- ☐ Trauma pre-alerts in general (1)
- ☐ Elderly/Silver trauma (2)
- ☐ Medical pre-alerts in general (3)
- ☐ Sepsis (4)
- ☐ Respiratory (5)
- ☐ Other (please state) (6) ____________________

End of Block: Block 1

Start of Block: Block 2

Section 2: Undertaking the pre-alert call
8. After a pre-alert decision is made by the crew on scene, who usually contacts the receiving ED?

○ Crew on scene (4)

○ Ambulance control centre (5)

○ Someone else in the ambulance service (please state who) (6)
____

○ Crew on scene for medical alerts and Trauma Desk for trauma alerts (9)

---

9. If you make the pre-alert call, what device do you mostly use to make the call?

○ Personal Mobile (1)

○ Work Mobile (2)

○ Ambulance Radio (3)

○ Other (please state) (4) ____

○ N/A I don't make the call (7)

---

10. Do you record the pre-alert in the patient notes?

	1 = Never	5 = Always

	1	2	3	4	5

Record pre-alert in patient notes ()	

11. How do you record the pre-alert in the patient record? (please tick one)

○ Free text only (4)

○ Free text plus tick box for pre-alert (5)

○ Tick box only (6)

○ Other (7) ____

12. How did you learn how to make a pre-alert call? (please select as many as apply)

☐ I was given specific training (1)

☐ Mentor/senior colleague (2)

☐ I learned to do it as I went along (3)

☐ Written guidelines (4)

☐ Other, please describe (5) ____

13 a. Have you ever had feedback on how you make pre-alert calls, or the cases you have pre-alerted? (tick all that apply)

- ☐ Yes - from managers/supervisors (1)
- ☐ Yes - from colleagues (2)
- ☐ Yes - from ED staff (3)
- ☐ Yes- from reporting systems e.g. Datix (4)
- ☐ No (5)
- ☐ Other (please state) (6) ____

---

13 b. If yes – did you find the feedback useful?

- ○ Yes (please briefly say why) (1) ____
- ○ No (please briefly say why) (2) ____
- ○ Sometimes useful (please briefly say why) (3) ____

End of Block: Block 2

Start of Block: Block 3

Section 3: Communicating with the Emergency Department

14. When making a pre-alert call, do you feel that ED staff

	1 = Never	5 = Always

	1	2	3	4	5

Listen to you and take the call seriously ()	
Listen without interrupting ()	
Make appropriate arrangements in the ED ()	

---

15. When you phone the ED, what format do you follow?

	1 - Never	5 = Always

	1	2	3	4	5

Use a predefined format e.g. ATMIST, ASHICE, SBAR ()	
Use a *different* pre-defined format, please state which ()	
Use the format that the receiving ED uses ()	
Provide observations but don't follow a fixed format ()	
Other (please state) ()	

---

16. Do you have anything else to add about pre-alerts?

____

____

____

____

____

End of Block: Block 3

Start of Block: Block 4

**Section 4: About you Thank you for taking the time to respond to our survey.
Please could you add some details about you so that we can understand how well our sample of respondents represents the wider staff in the ambulance service. We will not be able to identify you from this information.**

Which ambulance service do you work for?

- ○ YAS
- ○ WMAS
- ○ EMAS
- ○ LAS
- ○ NEAS
- ○ SCAS
- ○ SECAMB
- ○ EEAST
- ○ SWASFT
- ○ NWAS
- ○ Other (please state) ____
- ○ Private provider. Please state which private provider and which ambulance service trust you provide services to ____

What is your role?

- ◯ Paramedic (1)
- ◯ EMT (2)
- ◯ Specialist paramedic (3)
- ◯ Student paramedic (4)
- ◯ Other (please state) (7) ____

How long have you worked in this role?

- ◯ Less than 2 years (1)
- ◯ 2 - 5 years (2)
- ◯ 6 - 10 years (3)
- ◯ Longer than 10 years (4)

Are you....

- ◯ Female (1)
- ◯ Male (2)
- ◯ Non-binary (3)
- ◯ Other (please state) (4)
- ◯ Prefer not to say (5)

What is your ethnic group? (Choose one option that best describes your ethnic group or background)

- ◯ White (1)
- ◯ Asian or Asian British (2)
- ◯ Black or Black British (3)
- ◯ Chinese (4)
- ◯ Mixed (5)
- ◯ Any other Ethnic Group (please state) (6) ____
- ◯ Prefer not to say (7)

Do you wish to be entered into a prize draw to win a £50 Love2Shop voucher? Please note that if you select yes this will take you to a new survey link to enter your email address. This is so that we can keep your email address and survey responses separate.

◯ Yes

◯ No

End of Block: Block 4
