# Supplementary material for: Variation in ambulance pre-alert process and practice: cross-sectional survey of ambulance clinicians
Source: Emerg Med J. 2024 Dec 5;42(1):e213851. doi: 10.1136/emermed-2023-213851 (PMC11874312; doi:10.1136/emermed-2023-213851)
Supplement: online supplemental file 2 [file emermed-42-1-s002.pdf]

Table 1: Respondent and workforce characteristics

Role	Service 1 N (%)	Service 2 N (%)	Service 3 N (%)	Service 4 N (%)	Service 5 N (%)	Service 6 N (%)	Service 7 N (%)	Service 8 N (%)	Service 9 N (%)	Service 10 N (%)	All services N (%)
Paramedic	82 (60.3)	121 (53.8)	68 (42.2)	15 (26.3)	68 (64.2)	10 (31.3)	55 (57.3)	72 (51.1)	70 (70.0)	107 (43.9)	688 (51.5)
Specialist or senior paramedic	12 (8.8)	21 (9.3)	18 (11.2)	22 (38.6)	13 (12.3)	7 (21.9)	13 (13.5)	17 (12.1)	4 (4.0)	52 (21.3)	179 (13.8)
Student paramedic	9 (6.6)	27 (12.0)	12 (7.5)	7 (12.3)	6 (5.7)	2 (6.3)	5 (5.2)	14 (9.9)	7 (7.0)	14 (5.7)	103 (7.9)
EMT (Emergency Medical Technician, or equivalent)	29 (21.3)	49 (21.8)	60 (37.3)	13 (22.8)	14 (13.2)	11 (34.4)	20 (20.8)	35 (24.8)	9 (9.0)	67 (27.5)	307 (23.7)
Other	4 (2.9)	6 (2.7)	3 (1.9)	0	5 (4.7)	2 (6.3)	3 (3.1)	3 (2.1)	10 (10.0)	3 (1.2)	39 (3.0)
Total	136 (100)	225 (100)	161 (100)	57 (100)	106 (100)	32 (100)	96 (100)	141 (100)	100 (100)	244 (100)	1298 (100)
**Length of time in role**											
< 2 years	47 (34.6)	57 (25.3)	29 (18.0)	15 (26.3)	18 (17.0)	9 (28.1)	25 (26.0)	25 (17.7)	21 (21.0)	45 (18.4)	291 (22.4)
2 – 5 years	33 (24.3)	73 (32.4)	69 (42.9)	22 (38.6)	31 (29.2)	12 (37.5)	40 (41.7)	56 (39.7)		67 (27.5)	430 (33.1)
6 – 10 years	25	56	35	19	30	11	14	33	17	78	318

	(18.4)	(24.9)	(21.7)	(33.3)	(28.3)	(34.4)	(14.6)	(23.4)	(17.0)	(32.0)	(24.5)
>10 years	30<br>(22.1)	38<br>(16.9)	27<br>(16.8)	1<br>(1.8)	27<br>(25.5)	0	17<br>(17.7)	27<br>(19.1)	35<br>(35.0)	54<br>(22.1)	256<br>(19.7)
Total	136<br>(100)	225<br>(100)	161<br>(100)	57<br>(100)	106<br>(100)	32<br>(100)	96<br>(100)	141<br>(100)	100<br>(100)	244<br>(100)	1298<br>(100)
**Gender**											
Female	70<br>(51.5)	94<br>(41.8)	52<br>(32.3)	22<br>(38.6)	50<br>(47.2)	11<br>(34.4)	27<br>(28.1)	50<br>(35.5)	30<br>(30.0)	86<br>(35.2)	492<br>(37.9)
Male	63<br>(46.3)	123<br>(54.7)	107<br>(66.5)	30<br>(52.6)	53<br>(50.0)	20<br>(62.5)	66<br>(68.8)	85<br>(60.3)	67<br>(67.0)	154<br>(63.1)	768<br>(59.2)
Non-binary	2<br>(1.5)	2<br>(0.9)	1<br>(0.6)	2<br>(3.5)	1<br>(0.9)	0	0	2<br>(1.4)	0	2<br>(0.8)	10<br>(0.8)
Other	0	0	0	0	1<br>(0.9)	0	1<br>(1.0)	0	0	0	2<br>(0.2)
Prefer not to say	1<br>(0.7)	3<br>(1.3)	0	1<br>(1.8)	1<br>(0.9)	0	2<br>(2.1)	4<br>(2.8)	3<br>(3.0)	0	18<br>(1.4)
Total	136<br>(100)	225<br>(100)	161<br>(100)	55<br>(100)	106<br>(100)	31<br>(100)	96<br>(100)	141<br>(100)	100<br>(100)	242<br>(100)	1298<br>(100)
**Ethnicity**											
White	125<br>(91.9)	211<br>(93.8)	154<br>(95.7)	52<br>(91.2)	102<br>(96.2)	31<br>(96.9)	91<br>(94.8)	131<br>(92.9)	92<br>(92.0)	235<br>(96.3)	1224<br>(94.3)

Other ethnic groups combined	8 (5.9)	8 (3.2)	6 (3.6)	4 (7.0)	2 (1.9)	1 (3.1)	2 (2.0)	6 (4.3)	2 (2.0)	5 (2.0)	41 (3.1)
Prefer not to say	3 (2.2)	4 (1.8)	1 (0.6)	0	2 (1.9)	0	3 (3.1)	4 (2.8)	6 (6.0)	4 (1.6)	27 (2.0)
Total	136 (100)	225 (100)	161 (100)	57 (100)	106 (100)	32 (100)	96 (100)	141 (100)	100 (100)	244 (100)	1298 (100)

Table 2: Pre-alert practice by ambulance service

	**Service 1** **N (%)**	**Service 2** **N (%)**	**Service 3** **N (%)**	**Service 4** **N (%)**	**Service 5** **N (%)**	**Service 6** **N (%)**	**Service 7** **N (%)**	**Service 8** **N (%)**	**Service 9** **N (%)**	**Service 10** **N (%)**	**All services** **N (%)**
**Pre-alert frequency**											
Frequently e.g. several times a shift	17 (12.5)	17 (7.6)	8 (5.0)	5 (8.8)	5 (4.7)	4 (12.5)	5 (5.2)	2 (1.4)	2 (2.0)	27 (11.1)	92 (7.1)
Often e.g once a shift	39 (28.7)	56 (24.9)	48 (29.8)	13 (22.8)	28 (26.4)	8 (25.0)	25 (26.0)	32 (22.7)	13 (13.0)	75 (30.7)	337 (26.0)
Sometimes e.g. once or twice per week	58 (42.6)	118 (52.4)	82 (50.9)	24 (42.1)	51 (48.1)	14 (43.8)	50 (52.1)	75 (53.2)	50 (50.0)	110 (45.0)	632 (48.7)

Infrequently e.g once or twice per month	15 (11.0)	23 (10.2)	20 (12.4)	14 (24.6)	21 (19.8)	6 (18.8)	10 (10.4)	28 (19.9)	31 (31.0)	26 (10.7)	194 (14.9)
Other	7 (5.1)	10 (4.4)	3 91.9)	1 (1.8)	1 (0.9)	0	5 (5.2)	3 (2.1)	4 (4.0)	5 (2.0)	39 (3.1)
**Who contacts the ED**											
Crew on scene	89 (65.4)	108 (48.0)	126 (78.3)	14 (24.6)	10 (9.4)	15 (46.9)	80 (83.3)	122 (82.5)	91 (91.0)	56 (23.0)	711 (54.8)
Ambulance control centre	26 (19.1)	29 (12.9)	32 (19.9)	37 (64.9)	94 (88.7)	14 (43.8)	13 (13.5)	16 (11.3)	5 (5.0)	160 (65.6)	426 (32.8)
Someone else in the ambulance service	0	1 (0.4)	0	0	1 (0.9)	0	0	1 (0.7)	1 (1.0)	12 (4.9)	16 (1.2)
Crew on scene medical alerts and trauma desk for trauma alerts	20 (14.7)	84 (37.3)	3 (1.9)	6 (10.5)	1 (0.9)	2 (6.3)	1 (1.0)	2 (1.4)	3 (3.0)	16 (6.6)	138 (10.6)
**Device usually used to make the call**											
Personal mobile	68 (50.0)	138 (61.3)	125 (77.6)	23 (40.4)	19 (17.9)	12 (37.5)	11 (11.5)	110 (78.0)	82 (82.8)	19 (7.9)	607 (46.8)
Work mobile	49 (36.0)	19 (8.4)	26 (16.1)	21 (36.8)	13 (12.3)	15 (46.9)	8 (8.3)	19 (13.5)	3 (3.0)	55 (22.8)	228 (17.6)
Ambulance radio	15 (11.0)	66 (29.3)	10 (6.2)	11 (19.3)	58 (54.7)	4 (12.5)	72 (75.0)	11 (7.8)	10 (10.0)	133 (55.2)	390 (30.0)
Other	3	1	0	0	1	0	3	0	4	1	62

	(2.2)	(0.4)			(0.9)		(3.1)		(4.0)	(0.4)	(4.8)
**How pre-alert is recorded**											
Free text only	11 (8.1)	27 (12.0)	28 (17.4)	13 (22.8%)	15 (14.2)	4 (12.5)	5 (5.2)	10 (7.1)	18 (18.0)	34 (13.9)	165 (12.7)
Free text plus tick box for pre-alert	113 (83.1)	183 (81.3)	110 (68.3)	39 (68.4)	75 (70.8)	24 (75.0)	52 (54.2)	99 (70.2)	70 (70.0)	164 (67.2)	929 (71.6)
Tick box only	7 (5.1)	9 (4.0)	19 (11.8)	4 (7.0)	15 (14.2)	2 (6.3)	33 (34.4)	28 (19.9)	9 (9.0)	44 (18.0)	170 (13.1)
Other	3 (2.2)	4 (1.8)	4 (2.5)	0	0	1 (3.1)	4 (4.2)	4 (2.8)	3 (3.0)	2 (0.8)	25 (1.9)
**Given specific training on how to pre-alert**											
Yes	44 (32.4)	43 (19.1)	36 (22.4)	15 (26.3)	28 (26.4)	5 (15.6)	17 (17.7)	46 (32.6)	31 (31.0)	102 (41.8)	367 (28.3)
No	91 (66.9)	181 (80.4)	125 (77.6)	41 (71.9)	78 (73.6)	26 (81.3)	77 (80.2)	95 (67.4)	69 (69.0)	140 (57.4)	854 (65.8)
**Feedback (from ED or ambulance service)**											
Yes	77 (56.6)	123 (54.7)	88 (54.7)	50 (87.7)	36 (34.0)	22 (68.8)	43 (44.8)	68 (48.2)	44 (44.0)	144 (59.0)	
No	58 (42.6)	101 (44.9)	73 (45.3)	7 (12.3)	69 (65.1)	10 (31.3)	51 (53.1)	73 (51.8)	56 (56.0)	100 (41.0)	

Table 3: reasons for making a pre-alert call and factors impacting on pre-alert decisions

	All staff 1 = Never; 5 = Always				Paramedics 1=Never; 5=Always		Specialist/ senior paramedics 1=Never; 5=Always		Student paramedics 1=Never; 5=Always		EMTs 1=Never; 5=Always	
	**Mean (SD)**	**Median (IQR)**	**Always N (%)**	**Never N (%)**	**Mean (SD)**	**Median (IQR)**	**Mean (SD)**	**Median (IQR)**	**Mean (SD)**	**Median (IQR)**	**Mean (SD)**	**Median (IQR)**
**Reason for making a pre-alert**												
To inform ED staff of a potentially deteriorating patient	4.06 (0.933)	4 (4 – 5)	491 (38.2)	10 (0.8)	4.17 (0.926)	4 (4 – 5)	3.79 (0.89)	4 (3 – 4)	4.22 (0.83)	4 (4 – 5)	3.90 (0.96)	4 (3 – 5)
To give the ED time to make space in resus	3.79 (1.091)	4 (3 – 5)	401 (31.7)	31 (2.5)	3.90 (1.01)	4 (3 – 5)	3.47 (1.06)	4 (3 – 4)	3.77 (1.18)	4 (3 -5)	3.68 (1.10)	4 (3 – 5)
To ensure the patient is seen quicker on arrival	3.73 (1.177)	4 (3 – 5)	380 (31.9)	73 (6.1)	3.82 (1.20)	4 (3 – 5)	3.53 (1.08)	4 (3 – 4)	3.78 (1.14)	4 (3 – 5)	3.66 (1.14)	4 (3 – 5)
For advice about where to take the patient	2.50 (1.218)	2 (3 – 5)	55 (5.4)	268 (26.5)	2.16 (1.14)	2 (1 – 3)	3.15 (1.16)	3 (2 – 4)	2.45 (1.17)	2 (1 – 3)	2.80 (1.19)	3 (2 – 4)
**Sources of guidance used to help make pre-alert decisions**												
JRCALC	3.49 (1.13)	4 (2 – 4)	255 (19.6)	59 (4.5)	3.54 (1.16)	4 (3 – 4)	3.24 (0.97)	3 (3 – 4)	3.53 (1.23)	4 (3 – 5)	3.49 (1.06)	4 (3 – 4)
Local ambulance trust	3.75 (1.06)	4 (3 – 5)	338 (26.0)	36 (2.8)	3.81 (1.06)	4 (3 – 5)	3.62 (1.01)	4 (3 – 4)	3.94 (1.06)	4 (3 – 5)	3.65 (1.05)	4 (3 – 4)
Local hospital	3.10 (1.25)	3 (2 – 4)	140 (10.8)	149 (11.5)	2.99 (1.30)	3 (2 – 4)	3.42 (1.02)	3 (3 – 4)	3.23 1.31)	4 (2 – 4)	3.14 (1.19)	3 (2 – 4)

ACCE/RCEM	2.87<br>(1.27)	3<br>(2 – 4)	91<br>(7.0)	202<br>(20.6)	2.65<br>(1.30)	3<br>(1 – 4)	3.31<br>(1.11)	4<br>(3 – 4)	2.92<br>(1.3)	3<br>(2 – 4)	3.06<br>(1.19)	3<br>(2 – 4)
**Factors impacting on pre-alert decision making**												
Hospital transporting to	3.16<br>(1.147)	3<br>(2 – 4)	128<br>(9.9)	119<br>(9.2)	3.06<br>(1.18)	3<br>(2 – 4)	3.43<br>(0.95)	4<br>(3 – 4)	3.16<br>(1.18)	3<br>(2 – 4)	3.23<br>(1.12)	3<br>(3 – 4)
Distance from hospital	2.94<br>(1.21)	3<br>(2 – 4)	111<br>(8.6)	162<br>(12.5)	2.78<br>(1.21)	3<br>(2 – 4)	3.06<br>(1.19)	3<br>(2 – 4)	3.05<br>(1.25)	3<br>(2 – 4)	3.15<br>(1.18)	3<br>(2 – 4)
Anticipated Queue at the ED	3.00<br>(1.19)	3<br>(2 – 4)	100<br>(7.7)	151<br>(11.6)	2.92<br>(1.23)	3<br>(2 – 4)	3.22<br>(1.02)	3<br>(2.5 – 4)	2.98<br>(1.19)	3<br>(2 – 4)	3.10<br>(1.18)	3<br>(2 – 4)
Approaching end of shift	2.25<br>(1.33)	2<br>(1 – 3)	54<br>(4.2)	369<br>(28.4)	1.88<br>(1.28)	1<br>(1 – 3)	2.95<br>(1.12)	3<br>(2 – 4)	2.17<br>(1.32)	2<br>(1 – 3)	2.50<br>(1.28)	2<br>(1 – 4)
**Physiological criteria or specific conditions that trigger you to make a pre-alert call**												
Tachycardia >=131	3.54<br>(0.99)	4<br>(3 – 4)	234<br>(18.0)	19<br>(1.5)	3.54<br>(1.01)	4<br>(3 – 4)	3.45<br>(0.90)	3<br>(3 – 4)	3.71<br>(1.00)	4<br>(3 – 5)	3.53<br>(0.99)	4<br>(3 – 4)
Cardiac/Respiratory arrest	4.54<br>(0.90)	5<br>(5 – 5)	966<br>(74.4)	2<br>(0.2)	4.77<br>(0.68)	5<br>(5 – 5)	4.06<br>(1.04)	4<br>(3 – 5)	4.59<br>(0.95)	5<br>(5 – 5)	4.25<br>(1.04)	5<br>(4 – 5)
Unconscious with a GCS motor score of less than 4	4.43<br>(0.89)	5<br>(4 – 5)	829<br>(63.9)	4<br>(0.3)	4.62<br>(0.75)	5<br>(5 – 5)	3.98<br>(0.97)	4<br>(3 – 5)	4.49<br>(0.77)	5<br>(4 – 5)	4.20<br>(1.03)	5<br>(3 – 5)
Respiratory rate =25	3.68<br>(0.97)	4<br>(3 – 4)	299<br>(23.0)	12<br>(0.9)	3.68<br>(0.95)	4<br>(3 – 4)	3.60<br>(0.95)	4<br>(3 – 4)	3.86<br>(1.02)	4<br>(3 – 5)	3.66<br>(1.00)	4<br>(3 – 4)

Table 4: communication with the ED

	All staff 1 = Never; 5 = Always				Paramedics (1=Never; 5=Always)		Specialist/ senior paramedics (1=Never; 5=Always)		Student paramedics (1=Never; 5=Always)		EMTs (1=Never; 5=Always)	
	**Mean (SD)**	**Median (IQR)**	**Always N (%)**	**Never N (%)**	**Mean**	**Median (IQR)**	**Mean**	**Median (IQR)**	**Mean**	**Median (IQR**	**Mean**	**Median (IQR)**
**When making a pre-alert call to the ED, do you feel that ED staff**												
Listen to you and take the call seriously	3.31 (.90)	3 (3 – 4)	114 (8.8)	15 (1.2)	3.24 (0.87)	3 (3 – 4)	3.53 (0.88)	4 (3 – 4)	3.24 (0.91)	3 (3 – 4)	3.36 (0.94)	3 (3 – 4)
Listen without interrupting	3.09 (1.04)	3 (2 – 3)	115 (8.9)	59 (4.5)	3.04 (1.03)	3 (2 – 4)	3.11 (1.01)	3 (2 – 4)	2.83 (1.10)	3 (2 – 4)	3.26 (1.06)	3 (2 – 4)
Make appropriate arrangements in the ED	3.21 (0.94)	3 (3 – 4)	111 (8.6)	25 (1.9)	3.16 0.88)	3 (3 – 4)	3.38 (0.95)	3 (3 – 4)	3.20 (0.96)	3 (3 – 4)	3.20 (1.03)	3 (2 – 4)
**When you phone the ED, what format do you follow?**												
Use a predefined format e.g. ATMIST, ASHICE, SBAR	4.00 (0.98)	4 (3 – 5)	464 (35.7)	16 (1.2)	4.12 (0.97)	4 (4 – 5)	3.74 (0.94)	4 (3 – 4)	4.04 (0.96)	4 (3 – 5)	3.85 (0.98)	4 (3 – 5)
Use a *different* pre-defined format, please state	2.56 (1.31)	3 (1 – 4)	26 (2.0)	129 (9.9)	2.20 (1.36)	2 (1 – 3)	3.16 (1.0)	3 (3 – 4)	2.61 (1.48)	3 (1 – 4)	2.73 (1.19)	3 (2 – 4)
Use the format that the receiving ED uses	2.92 (1.31)	3 (2 – 4)	90 (6.9)	170 (13.1)	2.70 (1.39)	3 (1 – 4)	3.24 (1.10)	3 (2 – 4)	3.14 (1.34)	3 (2 – 4)	3.07 (1.16)	3 (2 – 4)

Provide observations but don't follow a fixed format	3.16<br>(1.24)	3<br>(2 – 4)	133<br>(10.2)	111<br>(8.6)	3.08<br>(1.32)	3<br>(2 – 4)	3.36<br>(1.12)	3<br>(3 – 4)	3.03<br>(1.25)	3<br>(2 – 4)	3.27<br>(1.13)	3<br>(3 – 4)
